# Biometric Evidence that Sexual Selection Has Shaped the Hominin Face

**DOI:** 10.1371/journal.pone.0000710

**Published:** 2007-08-08

**Authors:** Eleanor M. Weston, Adrian E. Friday, Pietro Liò

**Affiliations:** 1 Department of Biology, University College London, London, United Kingdom; 2 University Museum of Zoology, Downing Street, Cambridge, United Kingdom; 3 Computer Laboratory, University of Cambridge, William Gates Building, Cambridge, United Kingdom; University of St. Andrews, United Kingdom

## Abstract

We consider sex differences in human facial morphology in the context of developmental change. We show that at puberty, the height of the upper face, between the lip and the brow, develops differently in males and females, and that these differences are not explicable in terms of sex differences in body size. We find the same dimorphism in the faces of human ancestors. We propose that the relative shortening in men and lengthening in women of the anterior upper face at puberty is the mechanistic consequence of extreme maxillary rotation during ontogeny. A link between this developmental model and sexual dimorphism is made for the first time, and provides a new set of morphological criteria to sex human crania. This finding has important implications for the role of sexual selection in the evolution of anthropoid faces and for theories of human facial attractiveness.

## Introduction

This paper examines to what extent sex differences in human facial morphology are correlates of body size dimorphism, and differentiates components of the face that do not correspond to a model of ontogenetic scaling, or to the differential extension of common patterns of growth allometry [1]. In an evolutionary context, size-related shape differences in faces will evolve if body size is the main target of selection. However, such facial variation can serve to mask other distinctions between male and female faces that evolve independently of any selection pressure on body size. In studies of primates, in general where sexual dimorphism exists in the face, it arises principally through ontogenetic scaling, but some deviations from this pattern have been reported [2].

Modern humans are dimorphic in body size with men being approximately 15% greater on average than women in body mass [3]. Sexual dimorphism in facial size is generally apparent at approximately 14 years of age [4] and develops with the onset of puberty in association with the skeletal adolescent growth spurt [5]. Such size-related or ‘allometric’ facial variation is to be predicted, as it is associated with the prolonged growth (and hence delayed maturation) of the male face relative to that of the female [5]. Given the intense interest in human evolutionary biology, however, studies of sex differences in comparative growth of the human facial skeleton are surprisingly scarce [6]. Those that exist generally analyse only adults [6]–[10] or have focused on comparisons of lateral radiographs that characterise growth variation only in the midsagittal plane [11]–[13]. Commonly, in morphometric studies [14], [15] a representation of the sum of all the cranial variation is used to describe the ‘ontogenetic trajectory’. Consequently, determining from such studies which individual facial features do comply with predictions based on ontogenetic scaling [1], [16], and which do not, is difficult because knowledge of how the individual component parts of the face change during development is obscured. It is important to recognise that aspects of facial sexual dimorphism that do not comply with such ontogenetic predictions as these could be indicative of preferences for facial configurations that exert directional selection pressure on the evolution of human face-shape that is independent of any selection pressure on body size. Some departure from ‘ontogenetic scaling’ between the sexes in the craniofacial morphology of *Homo sapiens* has been recognised [11], [15] but the precise nature of, and mechanism underlying, this kind of morphological change has not been established.

The analysis here both includes an ontogenetic series of newborn to adult dry skulls, and also focuses on the individual components of the face and how they vary between the sexes. A similar comparative ontogenetic approach was undertaken previously to analyse the facial skeleton of the chimpanzee and gorilla [17] and this revealed that the male chimpanzee had a relatively broader, shorter face than that of the female, a finding that could not be explained by body size dimorphism alone. In contrast, variation in the male and female gorilla face was shown to be associated with ontogenetic scaling, and the type of facial distinction recognised in the chimpanzee was not present [17]. The work reported here sets out to clarify the ontogenetic basis of sexual variation in the human face and considers the functional, developmental and behavioural factors that may explain non-size-related facial distinctions in men and women.

## Results

From a comparison of male and female ontogenetic trajectories, calculated from dimensions recorded from the facial and basicranial skeleton of a native Southern African population of *Homo sapiens*, we show that most trait relationships do comply with a model of ontogenetic scaling (see comparisons represented by non-demarcated *P* values in Table 1, 2), but some do not comply with this model (see comparisons represented by demarcated significant *P* values in Table 1, 2)(see Material and Methods). Here, the relationships of upper facial height (FHT) and upper facial projection (FP) with a suit of cranial traits that characterise both the width and basicranial length of the face differ significantly between the sexes (Table 1 and Figure S1). ‘Facial projection (FP)’, although a distinct measure, essentially characterises a large component of ‘upper facial height (FHT)’ (Figure S1). As variation in anterior upper facial height (characterised by FHT and FP) is the dominant cause of the facial sexual dimorphism there is a knock-on effect on all traits correlated with it (see intercept comparisons in Table 1).

**Table 1:** Results from comparisons of *H. sapiens* male and female ontogenetic trajectories.

Cranium	1 BCL	2 FL	3 FHT	4 BZW	5 BJW	6 BMW	7 FPZ	8 FP	9 MW	10 OW
1 BCL	^–^	0.2035	0.0615	0.7483	0.6076	0.2178	0.0482	0.0397	0.1810	0.9682
2 FL	0.0850	–	0.3283	0.2129	0.6039	0.7803	0.3053	0.2067	0.6471	0.6494
3 FHT	0.0016*	0.0904	–	0.0150	0.1324	0.5520	0.6907	0.9181	0.7549	0.2078
4 BZW	0.4333	0.0692	0.0000**	–	0.1013	0.0492	0.0059*	0.0047*	0.0412	0.9307
5 BJW	0.4328	0.0684	0.0000**	0.9930	–	0.2403	0.1360	0.0612	0.2533	0.7170
6 BMW	0.7124	0.4358	0.0029*	0.1282	0.1000	–	0.7815	0.5173	0.8099	0.3896
7 FPZ	0.5590	0.2715	0.0003**	0.0667	0.0916	0.9073	–	0.4429	0.9804	0.3653
8 FP	0.0045*	0.1875	0.1571	0.0000**	0.0000**	0.0071*	0.0001**	–	0.7258	0.2174
9 MW	0.3988	0.7165	0.0173	0.0540	0.0783	0.6561	0.7066	0.0789	–	0.4419
10 OW	0.2367	0.0569	0.0002**	0.4408	0.4245	0.0933	0.0895	0.0006**	0.0699	–

P values (^*^ *P*≤0.01; ^**^ *P*≤0.001) from bootstrap tests comparing the major axis slope (above diagonal) and *y* intercepts (below diagonal) for male and female ontogenetic trajectories. Bold *P* values indicate level of significance after applying sequential Bonferroni correction. *R* values, major axis slope and *y* intercepts for trait relationships are given in Table S3. For trait definitions see below and Figure S1. In the cranium the intercept comparisons indicate that most of the trait relationships (non-demarcated) comply to a model of ontogenetic scaling but some deviate from this model: the relationship between upper facial height (FHT) and BCL, BZW, BJW, BMW, FPZ, OW and the relationship between facial projection (FP) and BCL, BZW, BJW, BMW, FPZ, OW, differ between the sexes. The cranial slope comparisons are not found to be significantly different between the sexes with the exception of the relationship between facial projection (FP and FPZ) and BZW (but note slope comparisons not significant after Bonferroni correction, see equivalent intercept comparisons).

BCL, basicranial length (basion [ba]–nasion [n]); FL, upper facial length (ba–prosthion [pr]); FHT, upper facial height (n–pr); BZW, bizygomatic width (zygion [zy]–zy); BJW, bijugal width (jugale [ju]–ju); BMW, bimaxillary width (zygomaxillare [zm]–zm); FPZ, facial projection (pr–zy); FP, facial projection (pr–frontomalare temporale [fmt]); MW, bimastoid width (mastoideale [ms]–ms); OW, orbital width (frontomalare orbitale [fmo]–maxillofrontale [mf]).

**Table 2:** Results from comparisons of *H. sapiens* male and female ontogenetic trajectories.

Mandible	1 ML	2 VL	3 I-CR	4 CHT	5 CRHT	6 CBD	7 GBD	8 CRBD	9 SHT	10 C-CR
1 ML	–	0.4001	0.0373	0.9140	0.0346	0.0615	0.1561	0.0312	0.2604	0.7786
2 VL	0.6839	–	0.1541	0.8052	0.1669	0.3505	0.4310	0.1282	0.5538	0.5099
3 I-CR	0.1904	0.3402	–	0.1927	0.9622	0.6110	0.4315	0.8972	0.3938	0.3009
4 CHT	0.1224	0.4707	0.7530	–	0.0302	0.1426	0.4125	0.1328	0.3941	0.6598
5 CRHT	0.0118	0.0401	0.2848	0.1149	–	0.5641	0.4461	0.7332	0.5042	0.0890
6 CBD	0.2812	0.4343	0.8579	0.9561	0.2537	–	0.8527	0.4470	0.8805	0.2003
7 GBD	0.0653	0.1295	0.6412	0.3894	0.7764	0.3472	–	0.2392	0.9256	0.2971
8 CRBD	0.1470	0.2438	0.7327	0.5734	0.5839	0.5397	0.7720	–	0.4172	0.1660
9 SHT	0.7914	0.6994	0.2484	0.3555	0.0537	0.3594	0.1176	0.1961	–	0.2792
10 C-CR	0.0368	0.0369	0.0306	0.0099*	0.0004**	0.0093*	0.0046*	0.0143	0.2007	–

P values (^*^ *P*≤0.01; ^**^ *P*≤0.001) from bootstrap tests comparing the major axis slope (above diagonal) and *y* intercepts (below diagonal) for male and female ontogenetic trajectories. Bold *P* values indicate level of significance after applying sequential Bonferroni correction. *R* values, major axis slope and *y* intercepts for trait relationships are given in Table S3. For trait definitions see below. In the mandible, the intercept and slope comparisons indicate that nearly all of the trait relationships comply with a model of ontogenetic scaling with the exception of the relationship between the distance between condyle and coronoid process of the mandible (C-CR) and CHT, CRHT, CBD, GBD, these differ between the sexes.

ML, length of mandible (gnathion [gn]–condylion laterale [cdl]); VL, ventral length of mandible (gn–gonion ventrale [go]); I-CR (infradentale [id]–coronion [cr]); CHT, posterior height of ramus (go–cdl); CRHT, height at coronoid process (go–cr); CBD, bi-condylar breadth (cdl–cdl), GBD, bi-gonial breadth (go–go); CRBD, bi-coronoidal breadth (cr–cr); SHT, symphysis height (gn–id); C-CR, distance between condyle and coronoid process (cdl–cr).

**Figure 1:**
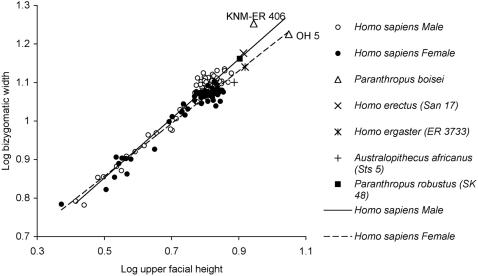
Male and female *H. sapiens* ontogenetic trajectories plotted with fossil hominin crania. The relationship between bizygomatic width (BZW) and upper facial height (FHT) shows a departure from ontogenetic scaling. Major axis slopes and 95% confidence intervals: male 0.7847 (0.74–0.83), female 0.6988 (0.65–0. 75). The FHT value for specimen KNM-ER 406 is a conservative estimate as the subnasal region is slightly damaged [28]. However, a small increase in FHT would align this cranium even more closely to the male ontogenetic trajectory.

**Figure 2:**
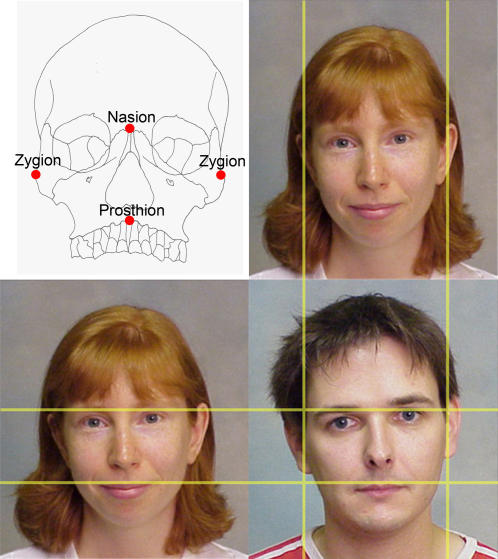
Skeletal craniofacial variables relate to facial appearance. We show that adult males have relatively shorter upper faces for their breadth compared to females (Table 1,2, Figure 1 and Figures S2 and S3). Lines superimposed on the pictures illustrate this facial dimorphism: vertical lines are positioned against the left and right zygion, and horizontal lines are positioned against the nasion and prosthion of the male face. In comparison to the female face, the male face is wider (represented by the distance between left and right point zygion) and the upper facial height (represented by the distance between point nasion and point prosthion) is approximately the same. The photographs are presented as taken, with identical camera-to-subject distance, and without rescaling, in order to represent the actual size of the faces.

The negative allometric relationship between bizygomatic width (BZW) and FHT exemplifies this sexual dimorphism (Figure 1). Young children possess shorter, broader faces relative to those of adults. However, a distinction between the sexes can also be observed that is linked to distinct male and female growth trajectories (Figure 1). Analysis of individual traits against age indicates that male and female growth trajectories diverge at puberty for BZW but not for FHT (Figures S2 and S3). This relationship of width-to-height of the upper face deviates from predictions based on ontogenetic scaling, as males (which are, on average, larger than females) have similar facial heights to females, whereas facial breadth is larger in the male: adult men have relatively shorter upper faces and adult women have relatively longer upper faces (Figure 2 and Figures S2, A and B). Hominin fossil crania with preserved facial skeletons reveal a similar type of sexual dimorphism (Figure 1).

In the mandible, most trait relationships comply with a model of ontogenetic scaling (Table 2). However, the relationships between the distance between the mandibular condyle and coronoid process (C-CR) and a suit of mandibular traits that characterise ramus height and mandibular breadth differ between the sexes (Table 2). C-CR is similar in male and female lower jaws, whereas the height of the mandible at the coronoid process (CRHT) is larger in the adult male (Figures S2, C and D). Volume changes during the growth and development of soft tissues in the human face have also been shown to differ at puberty in males and females [18]; the lower third of the male face (corresponding to the mandible) enlarging more than the middle and upper third [18]. We suggest that this pattern of facial dimorphism is not just linked with growth of the mandible but also reflects a reduction in the vertical growth of the anterior upper face.

## Discussion

Biomechanical and developmental aspects of variation in human craniofacial morphology impinge strongly on interpretations of facial sexual dimorphism. Each region of the face has one or more ‘counterparts’ requiring dynamic ‘fitting’ during growth [5], [19]. For example, a remodeling type of rotation of the mandible normally occurs during postnatal development. This has the effect of aligning and lengthening the ramus vertically in relation to the horizontal corpus, thus closing the gonial angle of the jaw. The vertical lengthening of the ramus is necessary to accommodate the posterior vertical growth of the nasomaxillary region of the face, and maintains the occlusal relationship between the maxillary and mandibular arches [5], [19]. In order to match the continued vertical growth of the midface and maintain facial balance, the ramus needs to lengthen vertically to a much greater extent than it broadens horizontally [5: 74–77]. Remodeling of the human maxilla during postnatal ontogeny usually increases the vertical dimension of the posterior upper face (‘vertical hyperplasia’) more than that of the anterior upper face (‘vertical hypoplasia’) resulting in the upward rotation of the nasomaxillary complex [20], [21].

Larger individuals (generally men) generate greater bite forces, and corresponding changes to facial form are necessary to maintain mechanical efficiency throughout ontogeny. It has been demonstrated that the occlusal force in long-faced adults is less than in individuals with normal vertical facial dimensions [22]. Reviews of orthodontic studies [23], [24] investigating the relationship between craniofacial morphology, muscle size and bite-force magnitude do not, however, provide a consensus linking a particular face type with increased mechanical efficiency; but measures reflecting relative differences between anterior and posterior vertical facial heights do nevertheless seem to be the most significant factors linked with optimal bite force [24]. However, if width-to-height facial dimorphism was associated with the functional demands of increased body size you would expect it to comply with an hypothesis of ontogenetic scaling, and not as demonstrated here, to deviate from it.

The sex-related differences in mandibular form reported here largely conform to biomechanical predictions. In the mandible, allometric growth is responsible for the narrowing (GBD, CBD, CRBD) and the posterior vertical lengthening (CHT, CRHT) of the jaw; the anterior posterior length (ML, VL) and anterior vertical height (SHT) increase isometrically (Figure S4) (see Materials and Methods). These biomechanical shape changes correlate with jaw size and conform to ontogenetic scaling predictions. In contrast, stabilising the degree of separation between the condyle and coronoid process (C-CR) in relation to ramus height (Table 2 and Figure S2D) conforms to a developmental ‘counterpart’ prediction: that the ramus should lengthen vertically more than it broadens horizontally to maintain facial balance [5].

A sex-specific association between the relative shortening of the upper face and extreme maxillary rotation through bone remodeling during ontogeny has not yet been demonstrated, but could provide a plausible non-mechanical explanation for this dimorphism. The same developmental model was proposed to explain the extreme thickness of the hard palate in *Paranthropus*, where palatal thickening was considered to be a simple by-product of a vertically expanded mandibular ramus [21], [25]. Prolonged positive allometric growth of the ramus and corresponding posterior vertical expansion of the upper face in men would determine the degree of anterior facial remodeling and the upward rotation of the nasomaxillary complex. The male nasal aperture would continue to expand vertically in response to the resorptive lowering of the anterior nasal floor [6] and would occupy a greater vertical proportion of the upper face relative to the nasal aperture of an adult female. A general absence of sexual dimorphism in the subnasal morphology of humans has been reported [26], [27]. The subnasal region is represented externally by the length of the nasoalveolar clivus (a component of FHT). The other components of FHT include the sagittal length of the nasal bones, a variable also characterised by an absence of sexual dimorphism [8], and the sexually dimorphic nasal aperture, which expands dorsally via the increased angulation of the nasal bones [6].

The recognition in humans of a link between sexual dimorphism and facial development is important, as it can be used as a tool to sex fossil hominin crania. In Figure 1 *Homo erectus* (Sangiran 17) and *Paranthropus robustus* (SK 48) align with the male ontogenetic trajectory and *Paranthropus boisei* (KNM-ER 406) is closer to the male slope than to that of the female. Conversely, *Australopithecus africanus* (Sts 5), *Homo ergaster* (KNM-ER 3733) and *Paranthropus boisei* (OH 5) align with the female ontogenetic trajectory (Figure 1). The sex allocation of *H. erectus* and *H. ergaster* skulls corroborates previous sex diagnoses, San 17 being a presumed male and ER 3733 a presumed female [28], [29]. However, the sex inferred from Figure 1 of the other hominin fossils does not always accord with previous deductions [26], [28], [30]. The two relatively large East African *Paranthropus* crania (KNM-ER 406 and OH 5) have previously both been considered males of the same species in spite of the notable difference in overall facial height [28]. Sts 5 was initially viewed as a female based on its small canine sockets, but more recently, based on a combination of size and morphological considerations, Sts 5 has been classified either as ‘sex indeterminate’ [26] or regarded as a small adult male [30].

The facial variation distinguishing *Paranthropus* crania OH 5 and KNM ER-406 corresponds to the same developmental predictions that underlie facial sexual dimorphism in modern humans (Table S1). The similarity of ontogenetic patterns of facial remodeling between *Paranthropus* and *H. sapiens* has been recognised before [31] but previously it has not been linked with sex. The large OH 5 cranium from Olduvai, originally assigned as a male [28], is here considered a female, based on the predicted correlates of a developmental model of posterior vertical facial hyperplasia and upward maxillary rotation (Table S1). This involves a radical change to current interpretations of hominin facial morphology both with regards to sexual dimorphism and to taxonomic affinities, but inferences of sex of fossil hominins should be enhanced through the inclusion of criteria based on a model of modern human facial development (Table S1).

Could the vertical modification of the anterior upper face in males and females be simply a by-product of developmental adjustments towards structural and functional balance? Or is there evidence to suggest that sexual selection, operating mainly through mate choice, has shaped the human face? Previously, ‘hormone markers’, singled out as cues that can affect judgements of male facial attractiveness [32], [33], have largely corresponded to regions of the face that grow allometrically, such as the lower jaw and browridges, and not necessarily to regions of the face that exhibit sex-specific size-independent variation, such as anterior upper facial height. A good example of a facial feature that is growth-related is cheekbone prominence: although male cheekbones are larger, female cheekbones appear more conspicuous than those of males, as the female nose, forehead and chin do not protrude to the same extent [5], [17]. Prominent cheekbones are attractive in both sexes but in females it is the relative anterior protrusion of the bone and amply overlying soft tissue [34] that defines them, as opposed to the degree of protrusion of the zygomatic bone laterally. In several studies of facial attractiveness [35]–[37] cheekbone prominence has been defined metrically by the ratio of the width of the face at the cheek-bones divided by the width of the face at the level of the mouth. The findings from these studies were not consistent as ‘cheekbone prominence’ in this context was found to be both greater in females [37]or greater in males [35], [36].

The sex-specific distinction (width-to-height of the upper face) reported here in a sample of modern humans, and potentially corresponding to facial dimorphism in other hominins, is quantifying different information, with width of the face across the cheekbones defined in relation to the height of the upper face and not defined in relation to the breadth of the lower jaw. The findings in this study suggest that, independent of any selection pressure on overall body size, it is upper facial height (and not facial breadth) that is the potential target of selection, as male upper faces are shorter than expected for their size. A divergence in the size of male and female traits usually occurs around male puberty at 12–14 years [4]. Our data confirm that sexual size dimorphism is present in most cranial traits (though to a variable degree) but is absent for FHT in post-pubescent individuals (Figure S3). The relationship between facial breadth across the cheekbones (BZW) and BCL (the usual proxy for skull size) is shown not to differ significantly between males and females, agreeing with predictions based on ontogenetic scaling, whereas the relationship between upper facial height and skull size (BCL) is shown to differ significantly between the sexes (Table 1, 2). This distinction, which separates facial variation hypothetically linked with sexual selective pressures acting on overall body size, from facial variation hypothetically linked with sexual selective pressures targeting a specific part of the face, is important as the latter should be able unambiguously to define adult male and female faces, whereas the former will fluctuate in accordance with variation in body size across human populations.

‘Good genes’ sexual selection models predict female preferences for exaggerated masculine traits [36] and yet–surprisingly–considerable variability in female preference for male faces has been documented both in respect of response to particular masculine facial traits and with regard to the hormonal state of the choosing individual across the menstrual cycle [32], [38]. It has been proposed that facial masculinity in humans could signal both benefits and costs to a potential partner [38]. These incongruent findings have been partly attributed to differences in the methods used to manipulate the masculinity of face images [32], [33], [39] but see [40]. Independent of the methods used to manufacture stimuli, the sex-specific distinction reported here (width-to-height of the upper face) is a manifestation of sex-specific facial development, and as such is a criterion worthy of evaluation in future studies of male/female face preferences.

**Figure 3:**
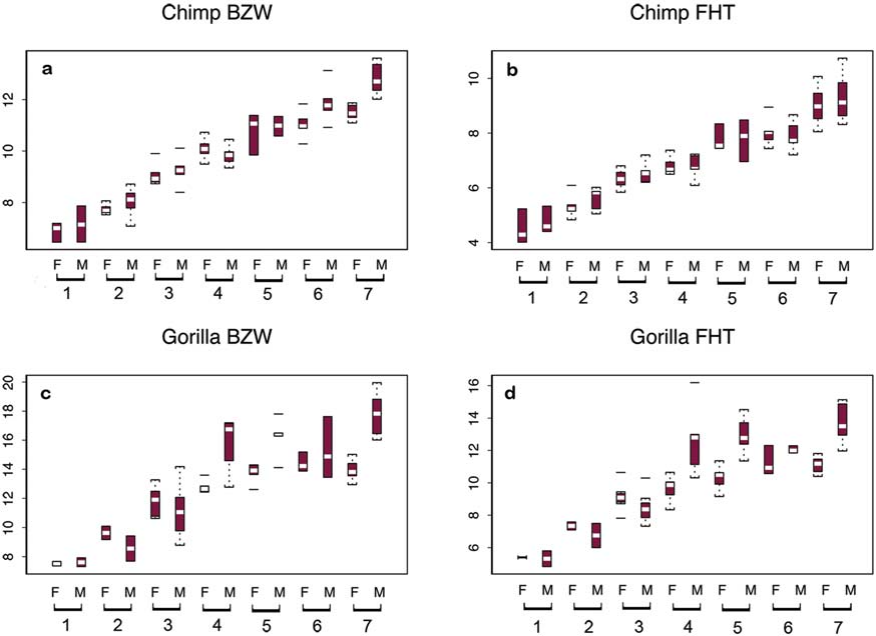
A comparison of male and female skeletal traits versus dental category (age class) for *Pan troglodytes* and *Gorilla gorilla.* For the chimpanzee, sexual size dimorphism is shown to be statistically significant in BZW in age classes 6 and 7 (A) and no significant sexual size dimorphism is evident in FHT for any age class (B). For the gorilla, sexual size dimorphism is evident in BZW (C) and FHT (D) for age classes 4, 5, and 7; BZW and FHT are sexually dimorphic in size as adults (age class 7). Age classes 1–7 plotted on the *x* axis; each class with females (F) plotted first and then males (M). Trait size on *y* axis (cm). The box plots indicate the median in white and the quantiles in colour. The dotted lines indicate the data range with outliers shown as isolated bars (for sample analysed see electronic Appendix in [17]).

If facial variation is considered across anthropoid primates, a similar type of facial width-to-height sexual dimorphism (though more exaggerated) has been noted in the common chimpanzee and some other primates [17]. The sex variation in the chimpanzee face [17] in contrast to that of humans (Table 1,2) is mostly indicated by slope and not intercept differences, and the degree of sexual dimorphism in the traits varies. However, the same relationship between width and height of the upper face in relation to body size (ontogenetic scaling) is shown to vary between males and females in humans and chimpanzees. Figure 3 provides a comparison of male and female cranial traits (BZW and FHT respectively) versus dental age category for *Pan troglodytes* and *Gorilla gorilla*. In the chimpanzee, though size dimorphism in facial breadth is evident by dental category 6, upper facial height is not significantly different in male and female adult chimpanzees (dental category 7), in spite of the larger male body size, analogous to the condition recorded in humans. In the gorilla, both variables exhibit size dimorphism by dental category 4 and both traits are dimorphic in adults. Chimpanzee and human facial sexual dimorphism with respect to FHT are developmentally similar, and potentially explained by the same constraints of maxillary rotation and lower jaw development. This type of facial dimorphism, expressed as an index, was shown to be negatively correlated with canine height dimorphism across a taxonomically mixed sample of anthropoid primates, suggesting that there could be some kind of trade off between facial dimorphism (signalling attractiveness) and canine dimorphism (signalling aggression) [17]. If *H. sapiens* is included in the primate sample (see Materials and Methods, Table S2 and Figure S5), a significant inverse relationship is evident between facial and canine sexual dimorphism, with modern humans exhibiting low canine sexual dimorphism but sexually dimorphic faces. If facial structure replaced canine size, or perhaps the general possession of a large anterior dentition, as a sexual selection signal in early hominins [17], it would suggest that facial attractiveness did, indeed, play a major role in shaping human evolution.

## Materials and Methods

### Morphometric analysis of modern skulls

Landmark co-ordinates were recorded using a MicroScribe G2 digitiser from 68 male and 53 female dry skulls (cranium and mandible digitised separately] of *H. sapiens* [native southern Africa population) from the Raymond Dart collection, University of Witwaterstrand, South Africa. Data from a cross-sectional, postnatal ontogenetic series of skulls (with age, sex and population group known from existing medical records) were taken (number of specimens per age class: neonate–1 year = 18; 2–10 years = 12; 11–16 years = 18; 17–20 years = 23; 21–24 years = 30; 25–30 years = 20). The sample is a cross section of different Southern African populations. This facilitated the analysis of complete male and female growth series given that sufficient numbers of juvenile specimens are not available for each individual population group. A previous study by De Villiers [41] of adult cranial variation concluded that the South African population groups exhibited the same order of variability, justifying the pooling of the samples for further analyses. The cranial and mandibular traits (inter-landmark distances) analysed are defined in Table 1,2. Landmarks used are as defined in Martin and Knußmann [42] and chord distances are as defined in the Koobi Fora Research Project IV [28]. Linear ontogenetic trajectories were calculated from logarithmically transformed variables using Model II regression, and the growth coefficients and *y* intercepts compared statistically using bootstrap tests for all relationships between variables (45 in total) [17], [43]. Basicranial length (nasion–basion), the usual proxy taken for skull size, is one of the variables included. For each test, 100000 bootstrap runs were performed six times on each sex (for mandibular and cranial traits separately) using both reduced major axis and major axis regression. The results were found to be comparable between bootstrap runs and for the different types of regression, thus only major axis data are presented (Table 1,2). The major axis confidence limits (Figure 1) were determined using a computer macro based on the computation given in Sokal and Rohlf [44]. In addition to the bootstrap analysis a sequential Bonferroni correction [35] was employed to determine the statistical significance of multiple comparisons, *P*≤0.001 (Table 1,2). The PC1 coefficients extracted from the covariance matrix of log-transformed ontogenetic data for each sex were calculated to depict the nature of allometric growth in the human mandible (Figure S4). In modern humans sexual dimorphism in facial size is evident at approximately 14 years of age according to the Bolton Standards [4]. Facial sexual dimorphism develops with the onset of puberty in association with the skeletal adolescent growth spurt (females 10–12 years; males 12–14 years) [4]. Size variability in these human data (before and after puberty) is statistically presented as a series of box plots recording male and female trait size per age class for all cranial traits (Figure S3). Figure 3 presents analogous growth data for *Pan troglodytes* and *Gorilla gorilla* for traits FHT and BZW (for sample analysed see Electronic Appendix in [17]).

### Fossil data

Chord distances for BZW and FHT for fossil hominin crania with preserved facial skeletons are taken from the Koobi Fora Research Project IV [28]. Estimated measures were not included, with the exception of KNM-ER 406 (see caption in Figure 1).

## Supporting Information

Figure S1 An illustration of the cranial landmarks and inter-landmark distances (traits) used in the analysis. Frontal aspect (A); lateral aspect (B). Cranial landmarks: ba = basion; fmo = frontomalare orbitale; fmt = frontomalare temporale; ju = jugale; mf = maxillofrontale; ms = mastoideale; n = nasion; pr = prosthion; zm = zygomaxillare; zy = zygion. Cranial traits: BCL, basicranial length (ba-n); FL, upper facial length (ba-pr); FHT, upper facial height (n-pr); BZW, bizygomatic width (zy-zy); BJW, bijugal width (ju-ju); BMW, bimaxillary width (zm-zm); FPZ, facial projection (pr-zy); FP, facial projection (pr-fmt); MW, bimastoid width (ms-ms); OW, orbital width (fmo-mf). Traits FHT and FP both represent measures of vertical facial height that combine an element of facial projection. Trait ‘FPZ’ though named facial projection characterises components of upper facial width and upper facial height.
(0.87 MB TIF)

Figure S2 A comparison of male and female skeletal traits versus age. Size dimorphism influenced by bimaturism (prolonged growth in the male relative to the female) evident in traits BZW and CRHT (A, C); no size dimorphism or indication of bimaturism evident in traits FHT and C-CR (B, D). Skeletal traits in cms defined in Table 1. Male (open circles), female (closed diamonds). Best-fit, least squares logarithmic curves: male [bold line], *r* ^2^ A-D, 0.926, 0.901, 0.807, 0.713; female (dashed line), *r* ^2^ A-D, 0.952, 0.891, 0.840, 0.809.
(1.37 MB TIF)

Figure S3 Box plots of human cranial trait size (cm) versus age classes. For each age class (0–1, 2–4, 6–10, 12–29), variation in female (F) and then male (M) trait size is shown. In the 12–29 age class sexual size dimorphism in BCL, BZW, BJW, FPZ is indicated by the separation of the median value in white and the quantiles shown in colour. For FL and OW these data (see 12–29 age class) show that male traits are larger than those of females but some overlap of the quantiles is evident. For BMW and MW, the male median value is larger than that of the female, but the quantiles overlap indicating a lower degree of sexual size dimorphism (not statistically significant across the pooled age class, 12–29). For FHT and FP, the median values are almost identical across all age classes and there is no significant sexual dimorphism exhibited for these traits. The dotted lines indicate the data range, with outliers shown as isolated bars. These size/age data suggest that the degree of size dimorphism evident in males post puberty (male puberty assumed to be around 12–14 years) is variable across different traits and absent for FHT.
(5.97 MB TIF)

Figure S4 Plot of PC1 coefficients depicting the nature of allometric growth in the human mandible. The posterior height of the mandible grows with positive allometry, the length of the mandible and the anterior vertical height grow isometrically and the breadth of the mandible grows with negative allometry. The lower jaw gets proportionately narrower and posteriorly taller with increased mandibular size. Markedly different male and female growth coefficients (e. g., C-CR) indicate a departure from ontogenetic scaling for that trait. The PC1 coefficients are extracted from the covariance matrices of log-transformed ontogenetic data for each sex. The isometric value (0.316) is determined by dividing 1 by the square root of the number of variables. Skeletal traits are defined in Table 1. The black diamonds are female and open circles male.
(2.49 MB TIF)

Figure S5 Inverse relationship between canine-height dimorphism and facial dimorphism in anthropoid primates including *H. sapiens*. (A) Raw data (F^1,13^ = 27.080 *p* = 0.0002 *r* = −0.822). (B) Phylogenetically independent contrasts (F^1,13^ = 13.125 *p* = 0.0031 *r* = −0.709). FDI (BZW dimorphism ratio/FHT dimorphism ratio); A. p, *Alouatta palliata*; A. s, *Alouatta seniculus*; At, *Ateles geoffroyi*; Ceb, *Cebus apella*; Cer, *Cercopithecus aethiops*; Col, *Colobus polykomos*; Gor, *Gorilla gorilla*; Hy, *Hylobates lar*; M. f, *Macaca fascicularis*; M. m, *Macaca mulatta*; Nas, *Nasalis larvatus*; Pan, *Pan troglodytes*; Pap, *Papio cynocephalus*; Pon, *Pongo pygmaeus*; Hom, *H. sapiens*.
(0.48 MB TIF)

Text S1 Supporting text. Methods associated with data presented in Figure S5.
(0.64 MB DOC)

Table S1 List of developmental predictions underpinning human facial sexual dimorphism. If a developmental model of posterior facial hyperplasia and upward maxillary rotation is adopted to explain sex-differences in human facial morphology, the following predictable correlates of male and female facial form would result (see first column ‘Developmental prediction’). If the same criteria are used to determine the sex of *Paranthropus boisei* cranial specimens, the data indicate that OH 5 is a female and KNM-ER 406 is a male. References cited in Table S1 are listed as a footnote.
(0.64 MB DOC)

Table S2 List of adult specimens and corresponding values used to calculate the *H. sapiens* Facial Dimorphism Index in Figure S5. All specimens included are 19 years of age or above to ensure completion of facial growth. FHT, upper facial height; BZW, bizygomatic width; M, male; F, female; A, Raymond Dart Collection Accession Number. Population group as defined in De Villers [1]. Interlandmark distances given in centimetres.
(0.64 MB DOC)

Table S3 Major axis slope (A) and *y* intercept values (B), and correlation coefficients (C) for cranial (Part I) and mandibular (Part II) *Homo sapiens* ontogenetic trajectories. Male values are given below the diagonal female values above the diagonal. Skeletal traits are defined in Table 1.
(0.64 MB DOC)
