# Supplementary figures and images for: Biometric Evidence that Sexual Selection Has Shaped the Hominin Face
Source: PLoS One. 2007 Aug 8;2(8):e710. doi: 10.1371/journal.pone.0000710 (PMC1937021; doi:10.1371/journal.pone.0000710)

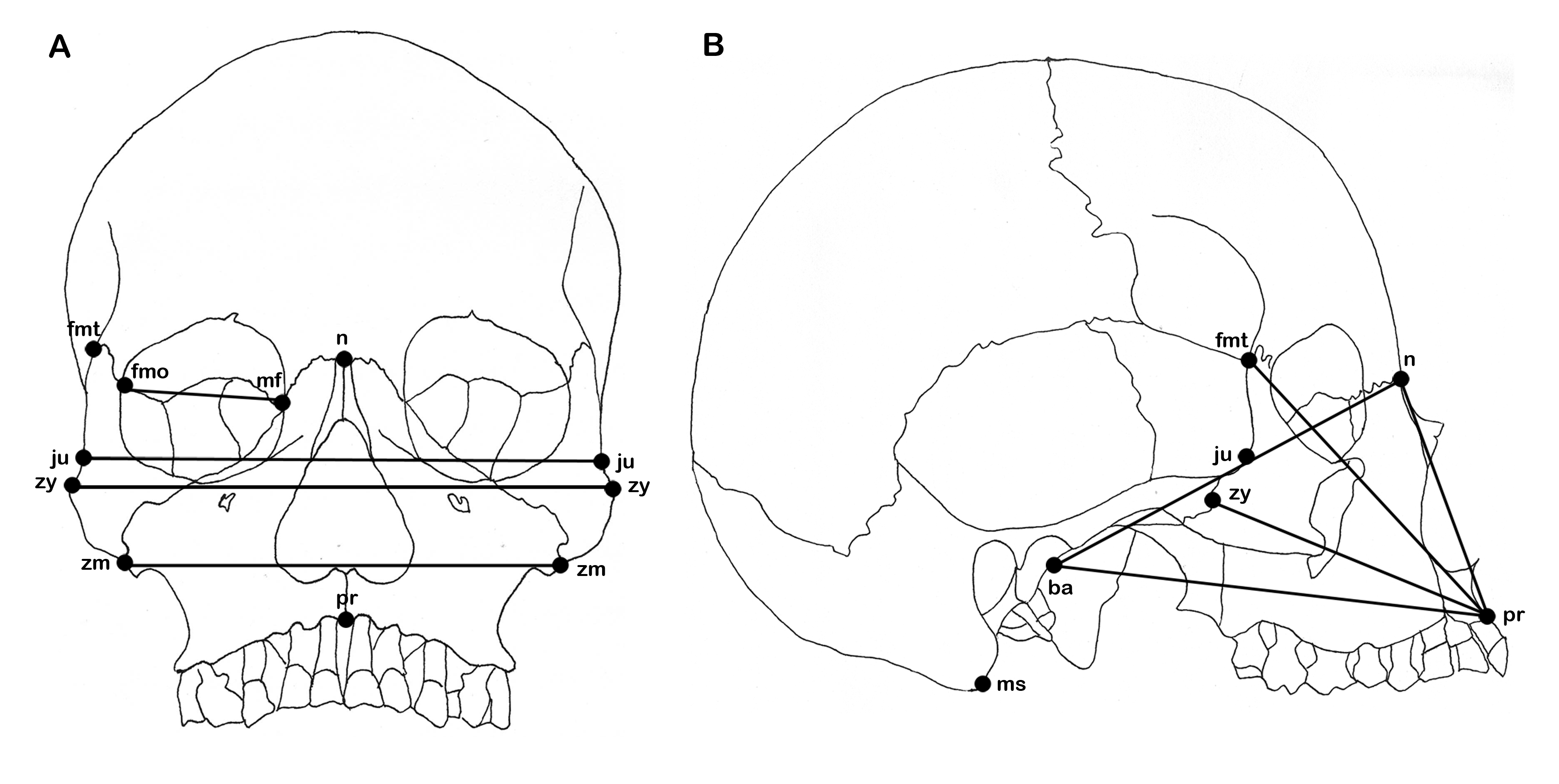

Supplement: Figure S1 — An illustration of the cranial landmarks and inter-landmark distances (traits) used in the analysis. Frontal aspect (A); lateral aspect (B). Cranial landmarks: ba = basion; fmo = frontomalare orbitale; fmt = frontomalare temporale; ju = jugale; mf = maxillofrontale; ms = mastoideale; n = nasion; pr = prosthion; zm = zygomaxillare; zy = zygion. Cranial traits: BCL, basicranial length (ba-n); FL, upper facial length (ba-pr); FHT, upper facial height (n-pr); BZW, bizygomatic width (zy-zy); BJW, bijugal width (ju-ju); BMW, bimaxillary width (zm-zm); FPZ, facial projection (pr-zy); FP, facial projection (pr-fmt); MW, bimastoid width (ms-ms); OW, orbital width (fmo-mf). Traits FHT and FP both represent measures of vertical facial height that combine an element of facial projection. Trait ‘FPZ’ though named facial projection characterises components of upper facial width and upper facial height. (0.87 MB TIF) [file pone.0000710.s001.tif]

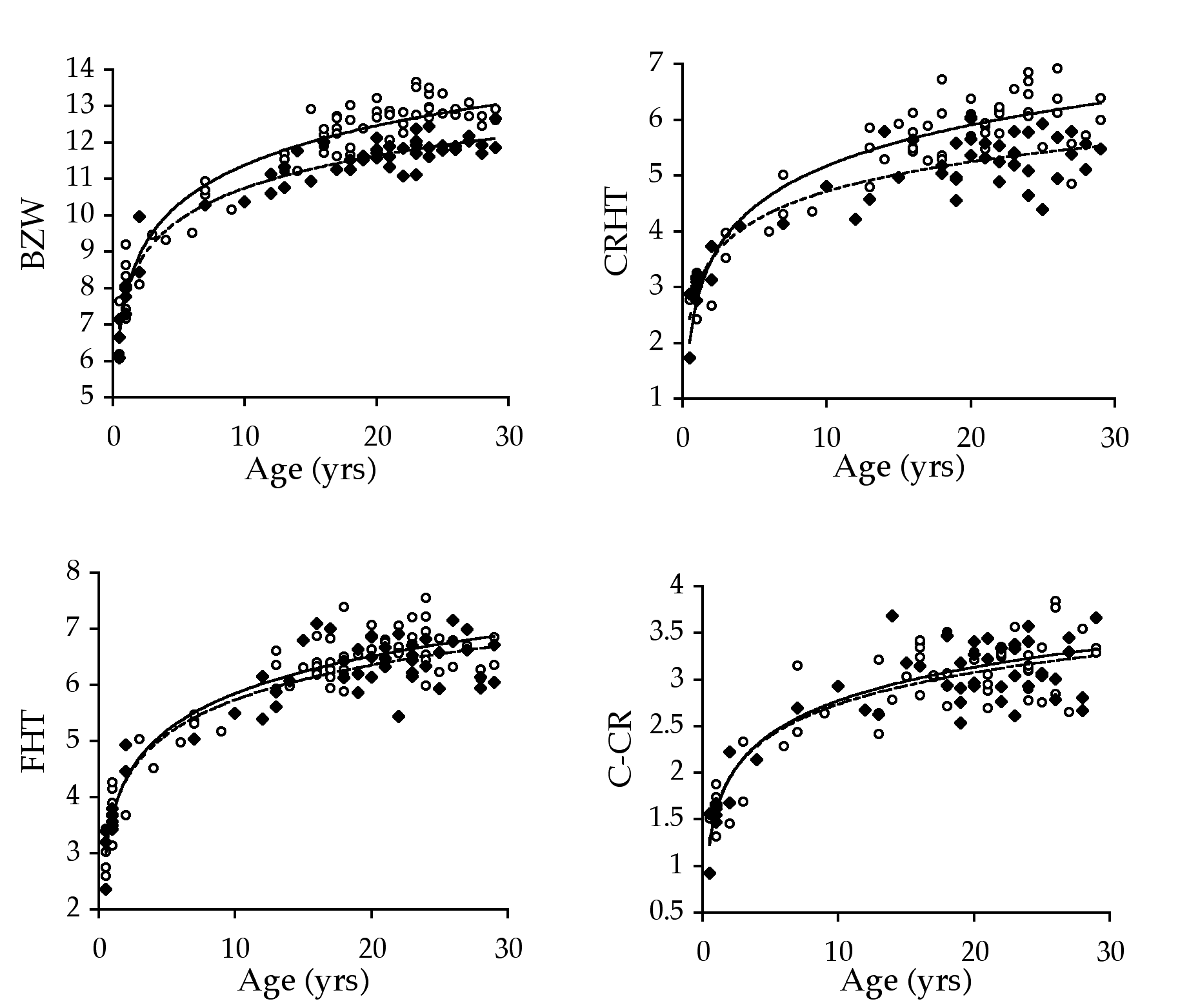

Supplement: Figure S2 — A comparison of male and female skeletal traits versus age. Size dimorphism influenced by bimaturism (prolonged growth in the male relative to the female) evident in traits BZW and CRHT (A, C); no size dimorphism or indication of bimaturism evident in traits FHT and C-CR (B, D). Skeletal traits in cms defined in Table 1. Male (open circles), female (closed diamonds). Best-fit, least squares logarithmic curves: male [bold line], r 2 A-D, 0.926, 0.901, 0.807, 0.713; female (dashed line), r 2 A-D, 0.952, 0.891, 0.840, 0.809. (1.37 MB TIF) [file pone.0000710.s002.tif]

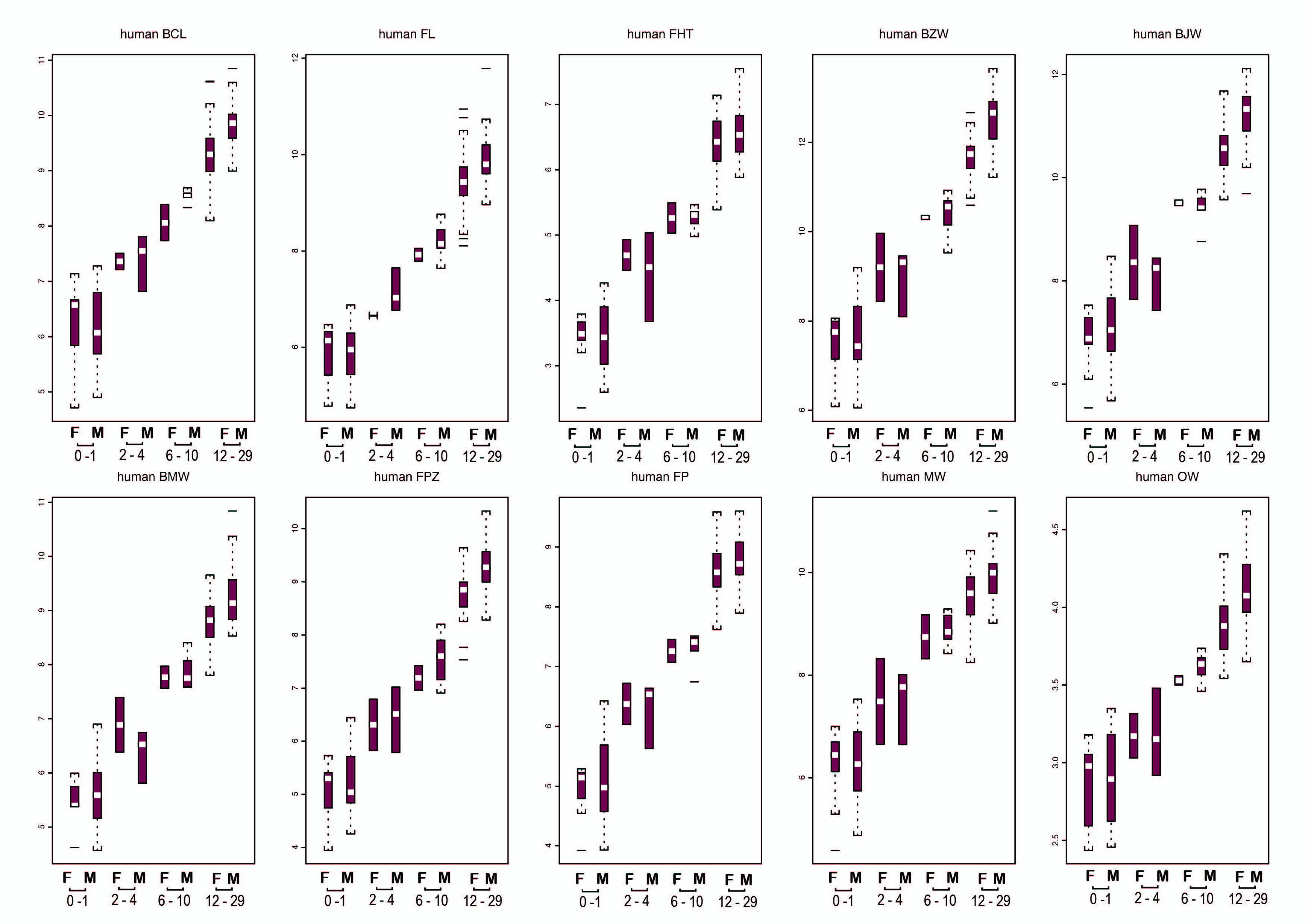

Supplement: Figure S3 — Box plots of human cranial trait size (cm) versus age classes. For each age class (0–1, 2–4, 6–10, 12–29), variation in female (F) and then male (M) trait size is shown. In the 12–29 age class sexual size dimorphism in BCL, BZW, BJW, FPZ is indicated by the separation of the median value in white and the quantiles shown in colour. For FL and OW these data (see 12–29 age class) show that male traits are larger than those of females but some overlap of the quantiles is evident. For BMW and MW, the male median value is larger than that of the female, but the quantiles overlap indicating a lower degree of sexual size dimorphism (not statistically significant across the pooled age class, 12–29). For FHT and FP, the median values are almost identical across all age classes and there is no significant sexual dimorphism exhibited for these traits. The dotted lines indicate the data range, with outliers shown as isolated bars. These size/age data suggest that the degree of size dimorphism evident in males post puberty (male puberty assumed to be around 12–14 years) is variable across different traits and absent for FHT. (5.97 MB TIF) [file pone.0000710.s003.tif]

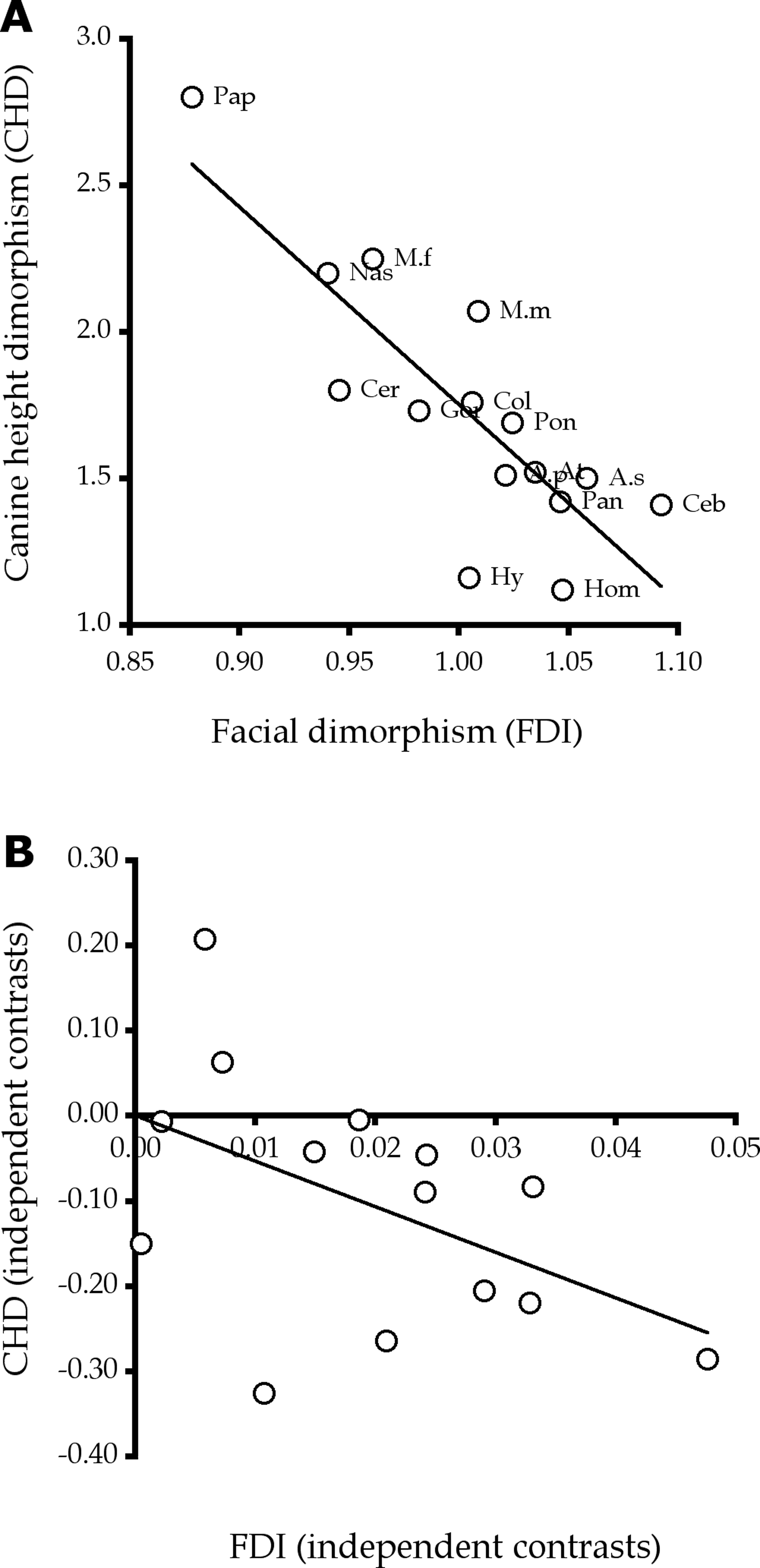

Supplement: Figure S5 — Inverse relationship between canine-height dimorphism and facial dimorphism in anthropoid primates including H. sapiens. (A) Raw data (F1,13 = 27.080 p = 0.0002 r = −0.822). (B) Phylogenetically independent contrasts (F1,13 = 13.125 p = 0.0031 r = −0.709). FDI (BZW dimorphism ratio/FHT dimorphism ratio); A. p, Alouatta palliata; A. s, Alouatta seniculus; At, Ateles geoffroyi; Ceb, Cebus apella; Cer, Cercopithecus aethiops; Col, Colobus polykomos; Gor, Gorilla gorilla; Hy, Hylobates lar; M. f, Macaca fascicularis; M. m, Macaca mulatta; Nas, Nasalis larvatus; Pan, Pan troglodytes; Pap, Papio cynocephalus; Pon, Pongo pygmaeus; Hom, H. sapiens. (0.48 MB TIF) [file pone.0000710.s005.tif]
