# Supplementary material for: Biometric Evidence that Sexual Selection Has Shaped the Hominin Face
Source: PLoS One. 2007 Aug 8;2(8):e710. doi: 10.1371/journal.pone.0000710 (PMC1937021; doi:10.1371/journal.pone.0000710)
Supplement: Text S1 — Supporting text. Methods associated with data presented in Figure S5. (0.64 MB DOC) [file pone.0000710.s006.doc]

|  |  |  |  |  |  |  |  |  |  |  |
| --- | --- | --- | --- | --- | --- | --- | --- | --- | --- | --- |
|  |  |  |  |  |  |  |  |  |  |  |
|  |  |  |  |  |  |  |  |  |  |  |
|  |  |  |  |  |  |  |  |  |  |  |
|  |  |  |  |  |  |  |  |  |  |  |
|  |  |  |  |  |  |  |  |  |  |  |
|  |  |  |  |  |  |  |  |  |  |  |
|  |  |  |  |  |  |  |  |  |  |  |
|  |  |  |  |  |  |  |  |  |  |  |
|  |  |  |  |  |  |  |  |  |  |  |
|  |  |  |  |  |  |  |  |  |  |  |
|  |  |  |  |  |  |  |  |  |  |  |
|  |  |  |  |  |  |  |  |  |  |  |
|  |  |  |  |  |  |  |  |  |  |  |
|  |  |  |  |  |  |  |  |  |  |  |
|  |  |  |  |  |  |  |  |  |  |  |
|  |  |  |  |  |  |  |  |  |  |  |
|  |  |  |  |  |  |  |  |  |  |  |
|  |  |  |  |  |  |  |  |  |  |  |
|  |  |  |  |  |  |  |  |  |  |  |
|  |  |  |  |  |  |  |  |  |  |  |
|  |  |  |  |  |  |  |  |  |  |  |

**Text S1**. Methods associated with data presented in Figure S5.

**Analysis of anthropoid facial sexual dimorphism**. The methods and most of the source data used in Figure S5 are published elsewhere [1]. The additional *H. sapiens* values for maxillary canine height dimorphism (CHD = 1.12) and facial dimorphism index (FDI = 1.05) were taken from specimens in the Raymond Dart Collection. Canine crown height (inter-landmark distance between tip of crown and cementum-enamel junction on mesio-buccal face) was recorded from the youngest individuals (n = 40) with permanent canines erupted, to minimise the effect of wear. The ‘facial dimorphism index’ was derived from the division of BZW dimorphism ratio (adult male mean divided by adult female mean, n = 30 of each sex) by the FHT dimorphism ratio [1]. The specimens and values used to calculate the facial dimorphism index (FDI) are given in Table S2. The adult sex ratios for FHT (1.038) and BZW (1.087) used here differ slightly from those calculated from the Dart Collection by De Villiers (FHT, 1.049; BZW, 1.062 [2]). Sample size and age composition will influence the sex ratio values reported in different studies (i.e., the inclusion of male individuals between 15 – 19 years of age will influence the size of the facial traits and the degree of sexual dimorphism as facial growth is incomplete). All specimens included in the calculation of sex ratios used in Figure 3 are 19 years of age or above to ensure completion of facial growth (Table S2). However, in this work and De Villers [41] the sex ratio for BZW is larger than the sex ratio for FHT.

1. Weston EM, Friday AE, Johnstone RA, Schrenk F (2004) Wide faces or large canines? The attractive versus the aggressive primate. Proc R Soc London Ser B Suppl, Biology Letters 271: S416-S419.
2. De Villiers H (1968) The skull of the South African Negro: a biometrical and morphological study. Johannesburg: Witwaterstrand University Press. 342 p.

|  |  |  |  |
| --- | --- | --- | --- |
|  |  |  |  |
|  |  |  |  |
|  |  |  |  |
|  |  |  |  |

. Interlandmark distances given in centimetres.

| |  |  |  | |  |  |  |  |  |  |  | | --- | --- | --- | --- | --- | --- | --- | --- | --- | --- | --- | |  |  |  | |  |  |  |  |  |  |  | |  |  |  | |  |  |  |  |  |  |  | |  |  |  | |  |  |  |  |  |  |  | |  |  |  |  | |  |  |  |  |  |  | |  |  |  |  | |  |  |  |  |  |  | |  |  |  |  | |  |  |  |  |  |  | |  |  |  |  | |  |  |  |  |  |  | |  |  |  |  | |  |  |  |  |  |  | |  |  |  |  | |  |  |  |  |  |  | |  |  |  |  | |  |  |  |  |  |  | |  |  |  |  | |  |  |  |  |  |  | |  |  |  |  | |  |  |  |  |  |  | |  |  |  |  | |  |  |  |  |  |  | |  |  |  |  | |  |  |  |  |  |  | |  |  |  |  | |  |  |  |  |  |  | |  |  |  |  | |  |  |  |  |  |  | |  |  |  |  | |  |  |  |  |  |  | |  |  |  |  | |  |  |  |  |  |  | |  |  |  |  | |  |  |  |  |  |  | |  |  |  |  | |  |  |  |  |  |  | |  |  |  |  | |  |  |  |  |  |  | |  |  |  |  | |  |  |  |  |  |  | |  |  |  |  | |  |  |  |  |  |  | |  |  |  |  | |  |  |  |  |  |  | |  |  |  |  | |  |  |  |  |  |  | |  |  |  |  | |  |  |  |  |  |  | |  |  |  |  | |  |  |  |  |  |  | |  |  |  |  | |  |  |  |  |  |  | |  |  |  |  | |  |  |  |  |  |  | |  |  |  |  | |  |  |  |  |  |  | |  |  |  |  | |  |  |  |  |  |  | |  |  |  |  | |  |  |  |  |  |  | |  |  |  |  | |  |  |  |  |  |  | |  |  |  |  | |  |  |  |  |  |  | |  |  |  |  | |  |  |  |  |  |  | |
| --- | --- | --- | --- | --- | --- | --- | --- | --- | --- | --- | --- | --- | --- | --- | --- | --- | --- | --- | --- | --- | --- | --- | --- | --- | --- | --- | --- | --- | --- | --- | --- | --- | --- | --- | --- | --- | --- | --- | --- | --- | --- | --- | --- | --- | --- | --- | --- | --- | --- | --- | --- | --- | --- | --- | --- | --- | --- | --- | --- | --- | --- | --- | --- | --- | --- | --- | --- | --- | --- | --- | --- | --- | --- | --- | --- | --- | --- | --- | --- | --- | --- | --- | --- | --- | --- | --- | --- | --- | --- | --- | --- | --- | --- | --- | --- | --- | --- | --- | --- | --- | --- | --- | --- | --- | --- | --- | --- | --- | --- | --- | --- | --- | --- | --- | --- | --- | --- | --- | --- | --- | --- | --- | --- | --- | --- | --- | --- | --- | --- | --- | --- | --- | --- | --- | --- | --- | --- | --- | --- | --- | --- | --- | --- | --- | --- | --- | --- | --- | --- | --- | --- | --- | --- | --- | --- | --- | --- | --- | --- | --- | --- | --- | --- | --- | --- | --- | --- | --- | --- | --- | --- | --- | --- | --- | --- | --- | --- | --- | --- | --- | --- | --- | --- | --- | --- | --- | --- | --- | --- | --- | --- | --- | --- | --- | --- | --- | --- | --- | --- | --- | --- | --- | --- | --- | --- | --- | --- | --- | --- | --- | --- | --- | --- | --- | --- | --- | --- | --- | --- | --- | --- | --- | --- | --- | --- | --- | --- | --- | --- | --- | --- | --- | --- | --- | --- | --- | --- | --- | --- | --- | --- | --- | --- | --- | --- | --- | --- | --- | --- | --- | --- | --- | --- | --- | --- | --- | --- | --- | --- | --- | --- | --- | --- | --- | --- | --- | --- | --- | --- | --- | --- | --- | --- | --- | --- | --- | --- | --- | --- | --- | --- | --- | --- | --- | --- | --- | --- | --- | --- | --- | --- | --- | --- | --- | --- | --- | --- | --- | --- | --- | --- | --- | --- | --- | --- | --- | --- | --- | --- | --- | --- | --- | --- | --- | --- | --- | --- | --- | --- | --- | --- | --- | --- | --- | --- | --- | --- | --- | --- | --- | --- | --- | --- | --- | --- | --- | --- | --- | --- | --- | --- | --- | --- | --- | --- | --- | --- | --- | --- | --- | --- | --- | --- | --- | --- | --- | --- | --- | --- | --- | --- | --- | --- | --- | --- | --- | --- | --- | --- | --- | --- | --- | --- | --- | --- | --- | --- | --- | --- | --- | --- | --- | --- | --- | --- | --- | --- | --- | --- | --- | --- | --- | --- | --- | --- | --- |
