# Supplementary material for: Biometric Evidence that Sexual Selection Has Shaped the Hominin Face
Source: PLoS One. 2007 Aug 8;2(8):e710. doi: 10.1371/journal.pone.0000710 (PMC1937021; doi:10.1371/journal.pone.0000710)
Supplement: Table S1 — List of developmental predictions underpinning human facial sexual dimorphism. If a developmental model of posterior facial hyperplasia and upward maxillary rotation is adopted to explain sex-differences in human facial morphology, the following predictable correlates of male and female facial form would result (see first column ‘Developmental prediction’). If the same criteria are used to determine the sex of Paranthropus boisei cranial specimens, the data indicate that OH 5 is a female and KNM-ER 406 is a male. References cited in Table S1 are listed as a footnote. (0.64 MB DOC) [file pone.0000710.s007.doc]

|  |  |  |  |  |  |  |  |  |  |  |
| --- | --- | --- | --- | --- | --- | --- | --- | --- | --- | --- |
|  |  |  |  |  |  |  |  |  |  |  |
|  |  |  |  |  |  |  |  |  |  |  |
|  |  |  |  |  |  |  |  |  |  |  |
|  |  |  |  |  |  |  |  |  |  |  |
|  |  |  |  |  |  |  |  |  |  |  |
|  |  |  |  |  |  |  |  |  |  |  |
|  |  |  |  |  |  |  |  |  |  |  |
|  |  |  |  |  |  |  |  |  |  |  |
|  |  |  |  |  |  |  |  |  |  |  |
|  |  |  |  |  |  |  |  |  |  |  |
|  |  |  |  |  |  |  |  |  |  |  |
|  |  |  |  |  |  |  |  |  |  |  |
|  |  |  |  |  |  |  |  |  |  |  |
|  |  |  |  |  |  |  |  |  |  |  |
|  |  |  |  |  |  |  |  |  |  |  |
|  |  |  |  |  |  |  |  |  |  |  |
|  |  |  |  |  |  |  |  |  |  |  |
|  |  |  |  |  |  |  |  |  |  |  |
|  |  |  |  |  |  |  |  |  |  |  |
|  |  |  |  |  |  |  |  |  |  |  |
|  |  |  |  |  |  |  |  |  |  |  |

**Table S1.** List of developmental predictions underpinning human facial sexual dimorphism. If a developmental model of posterior facial hyperplasia and upward maxillary rotation is adopted to explain sex-differences in human facial morphology, the following predictable correlates of male and female facial form would result (see first column ‘Developmental prediction’). If the same criteria are used to determine the sex of *Paranthropus boisei* cranial specimens, the data indicate that OH 5 is a female and KNM-ER 406 is a male. References cited in Table S1 are listed as a footnote.

| Developmental prediction | *Homo sapiens* | KNM-ER 406  (data from Wood 1991) [1] | OH 5  (data from Wood 1991) [1] |
| --- | --- | --- | --- |
| Superior Facial Index (FHT/BZW x 100) is greater in adult females than males | Female index = 55  Male index = 52  Mean values for traits used: n = 30.  Population: southern African  Sample: cross-sectional. | ‘Superior Facial 1’ index  = 49 | ‘Superior Facial 1’ index  = 67 |
| Ratio of nasal aperture height to FHT is greater in adult males. | Weston et al. (this paper): no data (rhinion, median most oral point of nasals, not recorded).  Kean & Houghton (1987)[2]: after puberty height of nasal cavity is greater in the male than the female  Wood et al. (1991) [3]: alveolar height (nasospinale – prosthion) and sagittal length of nasal bones (nasion – rhinion) not significantly sexually dimorphic in *H. sapiens.* | Nasal aperture height (rhinion – nasospinale)/FHT x 100  = 40  (nasal aperture ht = 36 mm) | Nasal aperture height (rhinion – nasospinale)/FHT x 100  = 30  (nasal aperture ht = 34 mm) |
| Alveolar maxillary prognathism is more pronounced in males than females. | Wood & Lynch (1996) (4): significant sexual dimorphism in alveolar prognathism has been documented in African & Romano-British modern human populations with the males being more prognathic than females. | Alveolar profile angle* = 45°  (more prognathic)  Alveolar height (nasospinale – prosthion) = 35 mm  * inclination of the alveolar process relative to the Frankfurt Horizontal in sagittal profile | Alveolar profile angle* = 63°  (more orthonathic)  Alveolar height (nasospinale – prosthion) = 42 mm  * inclination of the alveolar process relative to the Frankfurt Horizontal in sagittal profile |
| Ratio of posterior upper facial height to anterior upper facial (FHT) is greater in males than females | Braun et al. (1995) [5, 6]: in modern humans it has been shown that bite force is significantly larger in men than women and maximum bite force usually increases with an increasing ratio of posterior facial height to anterior facial height.  Sagittal cephalograms in orthodontist studies always include the mandible in measures of posterior and anterior facial height so comparable upper facial height indices are not available. | Posterior upper facial height^†^/FHT x100  = 68  ^†^chord distance between articular eminence and occlusal plane (McCollum 1994) [7] | Posterior upper facial height^†^/FHT x100  = 58  ^†^chord distance between articular eminence and occlusal plane (McCollum 1994) [7] |

1. Wood BA (1991) Koobi Fora Research Project IV: hominid cranial remains from Koobi Fora. Oxford: Clarendon Press. 492 p.
2. Kean MR, Houghton P (1987) The role of function in the development of human craniofacial form–a perspective. Anat Rec 218: 107-110.
3. Wood BA, Li Y, Willoughby C (1991) Intraspecific variation and sexual dimorphism in cranial and dental variables among higher primates and their bearing on the hominid fossil record. J Anat 174: 185-205.

1. Wood CG, Lynch JM (1996) Sexual dimorphism in the craniofacial skeleton of modern humans. In: Marcus LF, Corti M, Loy A, Naylor GJP, Slice DE, editors. Advances in Morphometrics. New York: Plenum Press. pp. 407-414.
2. Braun S, Bantleon H-P, Hnat WP, Freudenthaler JW, Marcotte MR et al. (1995) A study of bite force, part 1: relationship to various physical characteristics. Angle Orthod 65: 367-372.
3. Braun S, Bantleon H-P, Hnat WP, Freudenthaler JW, Marcotte MR et al. (1995) A study of bite force, part 2: relationship to various cephalometric measurements. Angle Orthod 65: 373-377.
4. McCollum MA (1994) Mechanical and spatial determinants of *Paranthropus* facial form. Am J Phys Anthrop 93: 259-273.

. Interlandmark distances given in centimetres.

| |  |  |  | |  |  |  |  |  |  |  | | --- | --- | --- | --- | --- | --- | --- | --- | --- | --- | --- | |  |  |  | |  |  |  |  |  |  |  | |  |  |  | |  |  |  |  |  |  |  | |  |  |  | |  |  |  |  |  |  |  | |  |  |  |  | |  |  |  |  |  |  | |  |  |  |  | |  |  |  |  |  |  | |  |  |  |  | |  |  |  |  |  |  | |  |  |  |  | |  |  |  |  |  |  | |  |  |  |  | |  |  |  |  |  |  | |  |  |  |  | |  |  |  |  |  |  | |  |  |  |  | |  |  |  |  |  |  | |  |  |  |  | |  |  |  |  |  |  | |  |  |  |  | |  |  |  |  |  |  | |  |  |  |  | |  |  |  |  |  |  | |  |  |  |  | |  |  |  |  |  |  | |  |  |  |  | |  |  |  |  |  |  | |  |  |  |  | |  |  |  |  |  |  | |  |  |  |  | |  |  |  |  |  |  | |  |  |  |  | |  |  |  |  |  |  | |  |  |  |  | |  |  |  |  |  |  | |  |  |  |  | |  |  |  |  |  |  | |  |  |  |  | |  |  |  |  |  |  | |  |  |  |  | |  |  |  |  |  |  | |  |  |  |  | |  |  |  |  |  |  | |  |  |  |  | |  |  |  |  |  |  | |  |  |  |  | |  |  |  |  |  |  | |  |  |  |  | |  |  |  |  |  |  | |  |  |  |  | |  |  |  |  |  |  | |  |  |  |  | |  |  |  |  |  |  | |  |  |  |  | |  |  |  |  |  |  | |  |  |  |  | |  |  |  |  |  |  | |  |  |  |  | |  |  |  |  |  |  | |  |  |  |  | |  |  |  |  |  |  | |  |  |  |  | |  |  |  |  |  |  | |  |  |  |  | |  |  |  |  |  |  | |  |  |  |  | |  |  |  |  |  |  | |
| --- | --- | --- | --- | --- | --- | --- | --- | --- | --- | --- | --- | --- | --- | --- | --- | --- | --- | --- | --- | --- | --- | --- | --- | --- | --- | --- | --- | --- | --- | --- | --- | --- | --- | --- | --- | --- | --- | --- | --- | --- | --- | --- | --- | --- | --- | --- | --- | --- | --- | --- | --- | --- | --- | --- | --- | --- | --- | --- | --- | --- | --- | --- | --- | --- | --- | --- | --- | --- | --- | --- | --- | --- | --- | --- | --- | --- | --- | --- | --- | --- | --- | --- | --- | --- | --- | --- | --- | --- | --- | --- | --- | --- | --- | --- | --- | --- | --- | --- | --- | --- | --- | --- | --- | --- | --- | --- | --- | --- | --- | --- | --- | --- | --- | --- | --- | --- | --- | --- | --- | --- | --- | --- | --- | --- | --- | --- | --- | --- | --- | --- | --- | --- | --- | --- | --- | --- | --- | --- | --- | --- | --- | --- | --- | --- | --- | --- | --- | --- | --- | --- | --- | --- | --- | --- | --- | --- | --- | --- | --- | --- | --- | --- | --- | --- | --- | --- | --- | --- | --- | --- | --- | --- | --- | --- | --- | --- | --- | --- | --- | --- | --- | --- | --- | --- | --- | --- | --- | --- | --- | --- | --- | --- | --- | --- | --- | --- | --- | --- | --- | --- | --- | --- | --- | --- | --- | --- | --- | --- | --- | --- | --- | --- | --- | --- | --- | --- | --- | --- | --- | --- | --- | --- | --- | --- | --- | --- | --- | --- | --- | --- | --- | --- | --- | --- | --- | --- | --- | --- | --- | --- | --- | --- | --- | --- | --- | --- | --- | --- | --- | --- | --- | --- | --- | --- | --- | --- | --- | --- | --- | --- | --- | --- | --- | --- | --- | --- | --- | --- | --- | --- | --- | --- | --- | --- | --- | --- | --- | --- | --- | --- | --- | --- | --- | --- | --- | --- | --- | --- | --- | --- | --- | --- | --- | --- | --- | --- | --- | --- | --- | --- | --- | --- | --- | --- | --- | --- | --- | --- | --- | --- | --- | --- | --- | --- | --- | --- | --- | --- | --- | --- | --- | --- | --- | --- | --- | --- | --- | --- | --- | --- | --- | --- | --- | --- | --- | --- | --- | --- | --- | --- | --- | --- | --- | --- | --- | --- | --- | --- | --- | --- | --- | --- | --- | --- | --- | --- | --- | --- | --- | --- | --- | --- | --- | --- | --- | --- | --- | --- | --- | --- | --- | --- | --- | --- | --- | --- | --- | --- | --- | --- | --- | --- | --- | --- | --- | --- | --- | --- | --- | --- | --- | --- | --- | --- | --- | --- |
