# Supplementary material for: Biometric Evidence that Sexual Selection Has Shaped the Hominin Face
Source: PLoS One. 2007 Aug 8;2(8):e710. doi: 10.1371/journal.pone.0000710 (PMC1937021; doi:10.1371/journal.pone.0000710)
Supplement: Table S2 — List of adult specimens and corresponding values used to calculate the H. sapiens Facial Dimorphism Index in Figure S5. All specimens included are 19 years of age or above to ensure completion of facial growth. FHT, upper facial height; BZW, bizygomatic width; M, male; F, female; A, Raymond Dart Collection Accession Number. Population group as defined in De Villers [1]. Interlandmark distances given in centimetres. (0.64 MB DOC) [file pone.0000710.s008.doc]

|  |  |  |  |  |  |  |  |  |  |  |
| --- | --- | --- | --- | --- | --- | --- | --- | --- | --- | --- |
|  |  |  |  |  |  |  |  |  |  |  |
|  |  |  |  |  |  |  |  |  |  |  |
|  |  |  |  |  |  |  |  |  |  |  |
|  |  |  |  |  |  |  |  |  |  |  |
|  |  |  |  |  |  |  |  |  |  |  |
|  |  |  |  |  |  |  |  |  |  |  |
|  |  |  |  |  |  |  |  |  |  |  |
|  |  |  |  |  |  |  |  |  |  |  |
|  |  |  |  |  |  |  |  |  |  |  |
|  |  |  |  |  |  |  |  |  |  |  |
|  |  |  |  |  |  |  |  |  |  |  |
|  |  |  |  |  |  |  |  |  |  |  |
|  |  |  |  |  |  |  |  |  |  |  |
|  |  |  |  |  |  |  |  |  |  |  |
|  |  |  |  |  |  |  |  |  |  |  |
|  |  |  |  |  |  |  |  |  |  |  |
|  |  |  |  |  |  |  |  |  |  |  |
|  |  |  |  |  |  |  |  |  |  |  |
|  |  |  |  |  |  |  |  |  |  |  |
|  |  |  |  |  |  |  |  |  |  |  |
|  |  |  |  |  |  |  |  |  |  |  |

|  |  |  |  |
| --- | --- | --- | --- |
|  |  |  |  |
|  |  |  |  |
|  |  |  |  |
|  |  |  |  |

**Table S2.** List of adult specimens and corresponding values used to calculate the *H. sapiens* Facial Dimorphism Index in Figure S5. All specimens included are 19 years of age or above to ensure completion of facial growth. FHT, upper facial height; BZW, bizygomatic width; M, male; F, female; A, Raymond Dart Collection Accession Number. Population group as defined in De Villers [1]. Interlandmark distances given in centimetres.

| | Acc. | Pop. | Age/ | | FHT | BZW | Acc. | Pop. | Age/ | FHT | BZW | | --- | --- | --- | --- | --- | --- | --- | --- | --- | --- | --- | | No. | Group | Yrs | | [M] | [M] | No. | Group | Yrs | [F] | [F] | | [M] | S. | [M] | |  |  | [F] | S. | [F] |  |  | |  | African |  | |  |  |  | African |  |  |  | | A155 | SHAN | 20 | 6.8564 | | 12.8571 | A1483 | TSWA | 19 | 5.8672 | 11.5890 | | A9 | VEND | 20 | 6.6326 | | 13.2281 | A1360 | SWAZ | 19 | 6.1916 | 11.6316 | | A554 | KALA | 20 | 7.0677 | | 12.6961 | A3997 | S.A.N. | 19 | 6.6324 | 11.5130 | | A578 | KALA | 21 | 6.8035 | | 12.8712 | A935 | ROLO | 20 | 6.4955 | 11.7951 | | A770 | KALA | 21 | 6.4050 | | 12.7517 | A863 | XOSA | 20 | 6.1385 | 11.5777 | | A550 | SOTO | 21 | 6.7540 | | 12.0737 | A3791 | ZULU | 20 | 6.8454 | 12.1144 | | A845 | ZULU | 22 | 6.6200 | | 12.8223 | A4069 | S.A.N. | 20 | 6.8736 | 11.7104 | | A500 | ZULU | 22 | 7.0566 | | 12.5069 | A3461 | SOTO | 21 | 6.3167 | 11.6111 | | A489 | SOTO | 22 | 6.6837 | | 12.2714 | A2492 | SOTO | 21 | 6.4727 | 11.8954 | | A847 | SOTO | 22 | 6.5649 | | 11.8448 | A3880 | S.A.N. | 21 | 6.6657 | 11.3179 | | A1264 | TSWA | 23 | 7.2085 | | 13.6587 | A787 | XOSA | 22 | 6.9108 | 11.8420 | | A3819 | SHAN | 23 | 6.7436 | | 12.7514 | A4035 | S.A.N. | 22 | 5.4352 | 11.0709 | | A752 | SOTO | 23 | 6.8468 | | 13.5228 | A3124 | ZULU | 23 | 6.7031 | 12.0360 | | A2369 | SOTO | 24 | 6.5375 | | 12.4416 | A1501 | ZULU | 23 | 6.4342 | 12.3680 | | A877 | SHAN | 24 | 6.4535 | | 12.9681 | A871 | TSWA | 23 | 6.1462 | 11.1096 | | A2648 | SWAZ | 24 | 7.5543 | | 13.3241 | A4060 | S.A.N. | 23 | 6.5337 | 11.7001 | | A1641 | SHIN | 24 | 7.2184 | | 13.5074 | A2849 | ZULU | 23 | 6.2193 | 11.9201 | | A783 | ZULU | 24 | 5.9871 | | 12.9344 | A2314 | XOSA | 24 | 6.8195 | 12.4448 | | A1487 | ROLO | 24 | 6.9593 | | 12.6876 | A1534 | SWAZ | 24 | 6.3297 | 11.8636 | | A702 | NDEB | 25 | 6.8305 | | 12.8033 | A644 | SOTO | 24 | 6.3299 | 11.6093 | | A748 | XOSA | 25 | 6.2315 | | 13.3379 | A2183 | SOTO | 25 | 5.9336 | 11.9009 | | A1350 | SHAN | 26 | 6.5108 | | 13.1352 | A745 | SOTO | 25 | 6.5778 | 11.7851 | | A1335 | ZULU | 26 | 6.7936 | | 12.7607 | A900 | SWAZ | 26 | 7.1444 | 11.8863 | | A1583 | SWAZ | 26 | 6.3189 | | 12.9184 | A1672 | XOSA | 26 | 6.7739 | 11.7960 | | A598 | POND | 27 | 6.6186 | | 12.7222 | A1499 | ZULU | 27 | 6.9872 | 12.0320 | | A1221 | GRIQ | 27 | 6.6957 | | 13.0991 | A1811 | TSWA | 27 | 6.6226 | 12.1796 | | A2030 | SHAN | 28 | 6.2793 | | 12.7246 | A3987 | S.A.N. | 28 | 5.9435 | 11.9260 | | A591 | XOSA | 28 | 6.1122 | | 12.4507 | A1549 | NDEB | 28 | 6.1387 | 11.6944 | | A3099 | SHAN | 29 | 6.8507 | | 12.9244 | A1468 | ZULU | 29 | 6.7158 | 12.6618 | | A382 | VEND | 29 | 6.3573 | | 12.6247 | A1653 | VEND | 29 | 6.0512 | 11.8535 | | Mean |  |  | 6.6851 | | 12.8407 |  |  |  | 6.4416 | 11.8145 | | Sex Ratio |  |  | 1.038 | | 1.087 |  |  |  |  |  | |
| --- | --- | --- | --- | --- | --- | --- | --- | --- | --- | --- | --- | --- | --- | --- | --- | --- | --- | --- | --- | --- | --- | --- | --- | --- | --- | --- | --- | --- | --- | --- | --- | --- | --- | --- | --- | --- | --- | --- | --- | --- | --- | --- | --- | --- | --- | --- | --- | --- | --- | --- | --- | --- | --- | --- | --- | --- | --- | --- | --- | --- | --- | --- | --- | --- | --- | --- | --- | --- | --- | --- | --- | --- | --- | --- | --- | --- | --- | --- | --- | --- | --- | --- | --- | --- | --- | --- | --- | --- | --- | --- | --- | --- | --- | --- | --- | --- | --- | --- | --- | --- | --- | --- | --- | --- | --- | --- | --- | --- | --- | --- | --- | --- | --- | --- | --- | --- | --- | --- | --- | --- | --- | --- | --- | --- | --- | --- | --- | --- | --- | --- | --- | --- | --- | --- | --- | --- | --- | --- | --- | --- | --- | --- | --- | --- | --- | --- | --- | --- | --- | --- | --- | --- | --- | --- | --- | --- | --- | --- | --- | --- | --- | --- | --- | --- | --- | --- | --- | --- | --- | --- | --- | --- | --- | --- | --- | --- | --- | --- | --- | --- | --- | --- | --- | --- | --- | --- | --- | --- | --- | --- | --- | --- | --- | --- | --- | --- | --- | --- | --- | --- | --- | --- | --- | --- | --- | --- | --- | --- | --- | --- | --- | --- | --- | --- | --- | --- | --- | --- | --- | --- | --- | --- | --- | --- | --- | --- | --- | --- | --- | --- | --- | --- | --- | --- | --- | --- | --- | --- | --- | --- | --- | --- | --- | --- | --- | --- | --- | --- | --- | --- | --- | --- | --- | --- | --- | --- | --- | --- | --- | --- | --- | --- | --- | --- | --- | --- | --- | --- | --- | --- | --- | --- | --- | --- | --- | --- | --- | --- | --- | --- | --- | --- | --- | --- | --- | --- | --- | --- | --- | --- | --- | --- | --- | --- | --- | --- | --- | --- | --- | --- | --- | --- | --- | --- | --- | --- | --- | --- | --- | --- | --- | --- | --- | --- | --- | --- | --- | --- | --- | --- | --- | --- | --- | --- | --- | --- | --- | --- | --- | --- | --- | --- | --- | --- | --- | --- | --- | --- | --- | --- | --- | --- | --- | --- | --- | --- | --- | --- | --- | --- | --- | --- | --- | --- | --- | --- | --- | --- | --- | --- | --- | --- | --- | --- | --- | --- | --- | --- | --- | --- | --- | --- | --- | --- | --- | --- | --- | --- | --- | --- | --- | --- | --- | --- | --- | --- | --- | --- | --- | --- | --- | --- | --- | --- | --- | --- |

1. De Villiers H (1968) The skull of the South African Negro: a biometrical and morphological study. Johannesburg: Witwaterstrand University Press. 342 p.
