# Supplementary material for: Biometric Evidence that Sexual Selection Has Shaped the Hominin Face
Source: PLoS One. 2007 Aug 8;2(8):e710. doi: 10.1371/journal.pone.0000710 (PMC1937021; doi:10.1371/journal.pone.0000710)
Supplement: Table S3 — Major axis slope (A) and y intercept values (B), and correlation coefficients (C) for cranial (Part I) and mandibular (Part II) Homo sapiens ontogenetic trajectories. Male values are given below the diagonal female values above the diagonal. Skeletal traits are defined in Table 1. (0.64 MB DOC) [file pone.0000710.s009.doc]

|  |  |  |  |  |  |  |  |  |  |  |
| --- | --- | --- | --- | --- | --- | --- | --- | --- | --- | --- |
|  |  |  |  |  |  |  |  |  |  |  |
|  |  |  |  |  |  |  |  |  |  |  |
|  |  |  |  |  |  |  |  |  |  |  |
|  |  |  |  |  |  |  |  |  |  |  |
|  |  |  |  |  |  |  |  |  |  |  |
|  |  |  |  |  |  |  |  |  |  |  |
|  |  |  |  |  |  |  |  |  |  |  |
|  |  |  |  |  |  |  |  |  |  |  |
|  |  |  |  |  |  |  |  |  |  |  |
|  |  |  |  |  |  |  |  |  |  |  |
|  |  |  |  |  |  |  |  |  |  |  |
|  |  |  |  |  |  |  |  |  |  |  |
|  |  |  |  |  |  |  |  |  |  |  |
|  |  |  |  |  |  |  |  |  |  |  |
|  |  |  |  |  |  |  |  |  |  |  |
|  |  |  |  |  |  |  |  |  |  |  |
|  |  |  |  |  |  |  |  |  |  |  |
|  |  |  |  |  |  |  |  |  |  |  |
|  |  |  |  |  |  |  |  |  |  |  |
|  |  |  |  |  |  |  |  |  |  |  |
|  |  |  |  |  |  |  |  |  |  |  |

|  |  |  |  |
| --- | --- | --- | --- |
|  |  |  |  |
|  |  |  |  |
|  |  |  |  |
|  |  |  |  |

. Interlandmark distances given in centimetres.

| |  |  |  | |  |  |  |  |  |  |  | | --- | --- | --- | --- | --- | --- | --- | --- | --- | --- | --- | |  |  |  | |  |  |  |  |  |  |  | |  |  |  | |  |  |  |  |  |  |  | |  |  |  | |  |  |  |  |  |  |  | |  |  |  |  | |  |  |  |  |  |  | |  |  |  |  | |  |  |  |  |  |  | |  |  |  |  | |  |  |  |  |  |  | |  |  |  |  | |  |  |  |  |  |  | |  |  |  |  | |  |  |  |  |  |  | |  |  |  |  | |  |  |  |  |  |  | |  |  |  |  | |  |  |  |  |  |  | |  |  |  |  | |  |  |  |  |  |  | |  |  |  |  | |  |  |  |  |  |  | |  |  |  |  | |  |  |  |  |  |  | |  |  |  |  | |  |  |  |  |  |  | |  |  |  |  | |  |  |  |  |  |  | |  |  |  |  | |  |  |  |  |  |  | |  |  |  |  | |  |  |  |  |  |  | |  |  |  |  | |  |  |  |  |  |  | |  |  |  |  | |  |  |  |  |  |  | |  |  |  |  | |  |  |  |  |  |  | |  |  |  |  | |  |  |  |  |  |  | |  |  |  |  | |  |  |  |  |  |  | |  |  |  |  | |  |  |  |  |  |  | |  |  |  |  | |  |  |  |  |  |  | |  |  |  |  | |  |  |  |  |  |  | |  |  |  |  | |  |  |  |  |  |  | |  |  |  |  | |  |  |  |  |  |  | |  |  |  |  | |  |  |  |  |  |  | |  |  |  |  | |  |  |  |  |  |  | |  |  |  |  | |  |  |  |  |  |  | |  |  |  |  | |  |  |  |  |  |  | |  |  |  |  | |  |  |  |  |  |  | |  |  |  |  | |  |  |  |  |  |  | |  |  |  |  | |  |  |  |  |  |  | |  |  |  |  | |  |  |  |  |  |  | |
| --- | --- | --- | --- | --- | --- | --- | --- | --- | --- | --- | --- | --- | --- | --- | --- | --- | --- | --- | --- | --- | --- | --- | --- | --- | --- | --- | --- | --- | --- | --- | --- | --- | --- | --- | --- | --- | --- | --- | --- | --- | --- | --- | --- | --- | --- | --- | --- | --- | --- | --- | --- | --- | --- | --- | --- | --- | --- | --- | --- | --- | --- | --- | --- | --- | --- | --- | --- | --- | --- | --- | --- | --- | --- | --- | --- | --- | --- | --- | --- | --- | --- | --- | --- | --- | --- | --- | --- | --- | --- | --- | --- | --- | --- | --- | --- | --- | --- | --- | --- | --- | --- | --- | --- | --- | --- | --- | --- | --- | --- | --- | --- | --- | --- | --- | --- | --- | --- | --- | --- | --- | --- | --- | --- | --- | --- | --- | --- | --- | --- | --- | --- | --- | --- | --- | --- | --- | --- | --- | --- | --- | --- | --- | --- | --- | --- | --- | --- | --- | --- | --- | --- | --- | --- | --- | --- | --- | --- | --- | --- | --- | --- | --- | --- | --- | --- | --- | --- | --- | --- | --- | --- | --- | --- | --- | --- | --- | --- | --- | --- | --- | --- | --- | --- | --- | --- | --- | --- | --- | --- | --- | --- | --- | --- | --- | --- | --- | --- | --- | --- | --- | --- | --- | --- | --- | --- | --- | --- | --- | --- | --- | --- | --- | --- | --- | --- | --- | --- | --- | --- | --- | --- | --- | --- | --- | --- | --- | --- | --- | --- | --- | --- | --- | --- | --- | --- | --- | --- | --- | --- | --- | --- | --- | --- | --- | --- | --- | --- | --- | --- | --- | --- | --- | --- | --- | --- | --- | --- | --- | --- | --- | --- | --- | --- | --- | --- | --- | --- | --- | --- | --- | --- | --- | --- | --- | --- | --- | --- | --- | --- | --- | --- | --- | --- | --- | --- | --- | --- | --- | --- | --- | --- | --- | --- | --- | --- | --- | --- | --- | --- | --- | --- | --- | --- | --- | --- | --- | --- | --- | --- | --- | --- | --- | --- | --- | --- | --- | --- | --- | --- | --- | --- | --- | --- | --- | --- | --- | --- | --- | --- | --- | --- | --- | --- | --- | --- | --- | --- | --- | --- | --- | --- | --- | --- | --- | --- | --- | --- | --- | --- | --- | --- | --- | --- | --- | --- | --- | --- | --- | --- | --- | --- | --- | --- | --- | --- | --- | --- | --- | --- | --- | --- | --- | --- | --- | --- | --- | --- | --- | --- | --- | --- | --- | --- | --- | --- | --- | --- | --- | --- | --- | --- | --- | --- | --- | --- | --- |

**Table S3.**  Major axis slope (A) and *y* intercept values (B), and correlation coefficients (C) for cranial (Part I) and mandibular (Part II) *Homo sapiens* ontogenetic trajectories. Male values are given below the diagonal female values above the diagonal. Skeletal traits are defined in Table 1.
